# Coping strategies among Iranian children with experience of Sarpol-e-Zahab earthquake: factor structure of children’s Coping Strategies Checklist-revision1 (CCSC-R1)

**DOI:** 10.1186/s40359-020-00456-8

**Published:** 2020-08-31

**Authors:** Zobeydeh Dehghan Manshadi, Hamid Taher Neshat Doost, Hooshang Talebi, Panos Vostanis

**Affiliations:** 1 Department of Psychology, University of Isfahan, Isfahan, Iran; 2 Department of Statistics, University of Isfahan, Isfahan, Iran; 3 Department of Neuroscience, University of Leicester, Leicester, UK

**Keywords:** Psychometric, Coping styles, Children, Culture, And trauma

## Abstract

**Background:**

Stress-related situations play a significant role in children’s lives and result in different reaction in children. Among various methods of evaluating the stressful environment of children, 54-item Children’s Coping Strategies Checklist-Revision1 (CCSC-R1) has been developed as one of the most powerful tools for assessing different aspects of coping in children. The purpose of the present study is to find the psychometric properties of Persian CCSC-R1 and to identify the coping strategies used by Iranian children.

**Method:**

Subjects included 401 female students aged between 9 and 13 who were subjected to the Sarpol-e-Zahab earthquake (in Northeast of Iran). Construct and convergence validities were examined with confirmatory factor analysis and correlated with Children’s Coping Behavior Questionnaire (CCBQ). Reliability was obtained by internal consistency. Using repeated analysis of variance, the status of coping strategies in children were achieved.

**Results:**

Confirmatory factor analysis showed a good model fit to the four-factor structure, active coping, distracting action/distraction, avoidance, and support seeking strategies. The results also demonstrated that there was a strong relationship between four-factors of CCSC-R1 including their subscales and CCBQ. Internal consistency (Cronbach’s Alpha) for the four dimensions were in the range of 0.76 to 0.88. The findings also showed that Iranian children use active coping, especially optimism, more than other strategies in order to deal with their stressful situations.

**Conclusions:**

It is concluded that CCSC-R1 is a valid and reliable instrument which could be employed for Iranian children. Furthermore, in the face of traumatic events, Iranian children acted same as people in individualistic cultures.

## Background

Stressful events in childhood cause numerous chronic diseases, psychiatric disorders, and mental health problems in an individual’s future life [1, 2]. Therefore, coping procedures play an essential role during childhood in facing with stressful situations. Compas et al. [3] defined coping as: “conscious, volitional efforts to regulate emotion, cognition, behavior, physiology, and the environment in response to stressful events or circumstances.” (p.89). Coping is mainly concerned with cognitive and behavioral attempts to conduct, reduce, or tolerate requests from external environments (e.g. family, friends, and society) or introverted environments (e.g. Intellectual/emotional conflict) [4].

Coping strategies are classified into multiple types in children and adults. In the first model, which is a general one, Lazarus and Folkman [5] categorized coping into two types of problem-focused and emotion-focused. In addition to these two coping strategies, Carver and Weintraub [6] proposed another influential factor called dysfunctional or avoidant coping. Ayers et al. [7] suggested four factors of coping strategies including active coping, avoidance, distraction, and social support. Also, Roger et al. [8] defined logical, detached, emotional, and avoidant coping in which logical and detached coping are generally employed as efficient strategies and emotional and the later ones are used as inefficient strategies. In contrast to the previous theories, Compas et al. [9] held the opinion that engagement (approach) and disengagement (avoidance) were the main coping strategies.

In the abovementioned models, measurement of coping strategies in children and adolescents during their stressful situations and traumatic events have assisted professionals in designing a proper therapeutic plan of coping skills. Therefore, different assessment tools such as questionnaires and interviews have attracted the attentions in this field. Self-report scales have been a widely used method, because of many advantages such as time-saving in measurement, simple data collected, and can be applied through different ways like oral/written or individual/group delivery [10]. Children’s Coping Strategies Checklist (CCSC) is one of the most important and commonly used questionnaires related to children which was proposed by Ayers et al. [7] and has 45 Likert-type questions. The CCSC is the most utilized questionnaire in traumatic events of children under 13 years old. The main feature of the scale is the measuring of coping strategies in general situations and specific events [10]. In their further investigation, Ayers and Sandler [11], introduced CCSC-R1 based on research in 1996 [12] which improved the form of CCSC. In this version, items and subscales were modified and increased from 45 to 54 and 10 to 13, respectively. Similar to CCSC, this scale is widely used for measuring coping strategies of children [13–16].

As numerous researchers have pointed out this questionnaire has psychometric proprieties in various versions such as English [11, 17–20], Dutch [21], Italian [22], and Arabic [23]. Psychometric characteristics (Cronbach’s alpha and the number of subscales) of all versions are summarized in Table 1. All of the factor analyses revealed 13 subscales, but in two different models: four-factor and five-factor. The English versions were noted as the Four-factor model and the Five-factor model was confirmed by Italian and Dutch versions. It seems that culture is involved in the difference of factor-models of American and European versions.

**Table 1:** Factors and Reliabilities in a different version of CCSC-R1

Subscale					Cronbach’s Alpha		
	Ayers, and Sandler	de Boo, and Wicherts	Gaylord-Harden	Morris, and Age	Camisasca et al.	Scott	Thorne et al.
	1999	2007	2008	2009	2012	2012	2013
	English version	Duch version	English version	English version	Italian version	English version	English version
	Sample: 356	Sample:436	Sample:235	Sample:65	Sample:727	Sample: 61	Sample: 506
	American	Duch	African-American	European-American, African-American, Latino, Biracial, Asian-American	Italian	African-American	Canadian
	Age range: 9 to 13	Age range: 8 to 13	Mean age: 10.37	Age range: 9 to 15	Age range: 9 to 14	Age range: 9 to 14	Age range: 8 to 11
***Active Coping Strategies***	0.88	−^1^	0.93	0.56	–	0.93	0.93
**a) Problem-Focused Coping**	0.80	0.85	NA	NA	0.80	0.87	NA
Cognitive Decision Making (CDM)	0.62	0.64	NA	NA	0.54	NA	NA
Direct Problem Solving (DPS)	0.61	0.68	NA	NA	0.59	NA	NA
Seeking Understanding (SU)	0.58	0.71	NA	NA	0.63	NA	NA
**b) Positive Cognitive Restructuring**	0.83	0.86	NA	NA	0.77	0.87	NA
Positive Thinking/or Positivity (POS)	0.62	0.66	NA	NA	0.56	NA	NA
Control (CON)	0.66	0.75	NA	NA	0.60	NA	NA
Optimism Thinking (OPT)	0.66	0.70	NA	NA	0.61	NA	NA
***Distraction Strategies***	NA	0.77	0.82	0.58	0.77	0.77	0.82
Distracting Actions (DA)	NA	0.62	NA	NA	0.62	NA	NA
Physical Release of Emotions (PRE)	NA	0.66	NA	NA	0.66	NA	NA
***Avoidance Strategies***	0.65	0.72	0.77	0.76	0.69	0.83	0.77
Avoidant Actions (AVA)	0.43	0.51	NA	NA	0.53	NA	NA
Repression (REP)	0.43	0.50	NA	NA	0.49	NA	NA
Wishful Thinking (WISH)	0.62	0.72	NA	NA	0.52	NA	NA
***Support-Seeking Strategies***	0.86	0.88	0.86	0.82	0.83	0.91	0.86
Support for Actions (SUPA)	0.74	0.77	NA	NA	0.72	NA	NA
Support for Feeling (SUPF)	0.79	0.82	NA	NA	0.74	NA	NA

1. Not mentioned as a category

The preference for a coping strategy is not merely the product of personal attitudes (such as personality traits), rather it is due to the specific interaction between the individual and his/her environment [24]. Hence, culture seems to be a determinant of the difference between factor models of American and European versions. The cultural orientation of countries can be considered as one of the most important reasons for the difference in people’s coping strategies [25]. Most studies are based on well-known cultural patterns of individualism and collectivism [26]. Accordingly, in collectivist cultures, individuals feel a deep sense of belonging to their group and community. The collectivist culture’s emphasis is on honesty and loyalty to the group and behavior of people is measured by the rules, goals, and values of the group. On the alternative side, there are individualistic cultures in which the desires and emotions of the individual have priority over those of the group and society. In these communities, there is freedom for individuals to do what they prefer. The two aspects of individualism-collectivism affect a wide range of behaviors, such as coping styles. People in collectivist cultures are interested in intragroup cohesion and harmony. They use strategies such as projection, acceptance, perseverance, religion [24], and internally targeted control to adapt to the environment so as not to disturb other group members or harm intragroup relationships [27]. Action-oriented problem-solving strategies are preferred in European and North American cultures, whereas people in Asian culture prefer to use task-oriented strategies such as cognitive-appraisal and not to use direct action-based coping methods such as self-disclosure or direct confrontation [28].

This cultural difference is also seen in adolescences. When faced with problems, Asian adolescents tend to use emotion-focused coping, and in European countries teens use problem-focused strategies [29]. Persike and Seiffge-Krenke [30] selected 10,941 adolescents with a mean age of 15.18 (girls and boys) from 20 countries (Western, Eastern/Asian, and Southern) in an extensive study to examine coping styles. The Western region used the most negotiating, seeking support, and emotional outlet styles. Generally, women in all regions compared to men preferred to use negotiating and seeking support strategies in stressful situations. Two withdrawal and denial strategies consist of one-fifth of all the strategies used by adolescents in different regions of the world. Mihalca et al. [31] found maladaptive coping strategies are used more among Moldavian teens compared to Romanian teens. The most common way of coping among Japanese, Chinese, and Korean immigrant students is social support networks. Also, Korean students are more likely to use religious practices when faced with problems [32]. However, few studies have been conducted on the relation of culture and coping strategy in the Middle East. Braun-Lewensohn et al. [33] compared the coping strategies of the 303 Israeli Jewish and Arab teens who were exposed to rocket attacks during the Second Lebanese War. They showed that both Jewish and Arab adolescents often use “problem-solving coping strategies”, while “reference to others” and “non-productive” coping strategies were most often used by Arab adolescents. Shirazi et al. [34] also demonstrated that Indian men and women had higher scores on avoidance focused coping strategies than Iranians. Also, this strategy is more frequently reported in Iranian men than women.

Since cultures affect adolescents’ adaptation and few studies have examined the coping strategies used in the Middle East, identification of such strategies through examining psychometric features of the widely used CCSC-R1 questionnaire can be helpful for Iranian children to use and better understand the impact of culture. The difference between versions of CCSC-R1 questionnaire with different numbers of factors can also be found. Accordingly, the present study attempts to investigate the psychometric traits of CCSC-R1 questionnaire and coping strategies used by Iranian adolescents in the time of natural earthquake trauma.

## Methods

In the present study, descriptive type of cross-sectional study was applied. The Banville method [35] was used to translate CCSC-R1. First, the questionnaire was translated into Persian language. Then, to compare content validity and concept equality with the original version, three clinical psychologists were requested to evaluate and correct the Persian version of CCSC-R1. Afterward, the edited questionnaire was given to an expert fluent in English to back translate the items. The new version was compared with the original one to check whether it had the same concept or not. The reliability was obtained by asking 20 students, who were the same as the research sample in the pilot phase, to answer the questions. The Internal Consistency (Cronbach’s alpha) was confirmed with the value 0.80 which is within an acceptable range.

### Psychometric testing

#### Children’s coping strategies checklist-revision

The reversion version of the CCSC is the CCSC-R1. Developed by Ayers and Sandler [11], The CCSC-R1 included 54 items self-report inventory which is suitable for 9–13-year-old children and measure 13 subscales of coping strategies with stress in four dimensions: Active Coping Strategies, Distraction Strategies, Avoidance Strategies, Support-Seeking Strategies. The CCSC-R1 ascertain the amount of the children attempts use to control their emotion, though, behavior, physiology, and the environment when they face with stressful events of circumstances. All of the queries started with “If I have a problem”. Children answered the questions in Likert form from 1 (never) to 4 (always). A high score on a subscale is indicative of use of that strategy. This tool had adequate validity and reliability in Ayers et al.’s study [11].

#### Children’s coping behavior questionnaire

This scale was produced by Hernandez in 2008 [36] and contained 57 statements and three subscales (deviation from the problem, coping with the problem, destructive coping). CCBQ is appropriate for 10–16-year-old children. In the Hernandez study [36], adequate psychometric properties were reported. Cronbach’s alpha ranged between 0.87 and 0.93. Fallahi et al. [37] reviewed the psychometric properties of CCBQ in 300 students in Northern Iran (Guilan). Findings, based on factor analysis, showed that three factors (diversion coping, ameliorative coping, and destructive coping) explained 60.9 percentage of the total variance. Satisfaction correlations between CCBQ and Inventory Children Anxiety Trait-S demonstrated appropriate concurrent validity. Cronbach’s alpha and test-retest were between 0.74 and 0.91.

### Data collection procedure

This research was performed as a developmental study using a cross-sectional design. Consent was initially obtained from Education Department (for public schools), principals of schools, and parents of children. Based on Comrey and Lee’s [38] suggestion for selecting a good sample, 500 students aged 9–13 years old, who were present in Sarpol-e-Zahab (in Kermanshah Province in Iran) earthquakes on November 12, 2017 and November 26, 2018, were selected by cluster sampling method. For this purpose, in order to select the required sample, seven elementary schools were randomly selected after preparing a complete list of all girls’ primary schools. In the next step, a list of fourth, fifth and sixth grades was prepared and three classes were selected from each school. The questionnaires were completed in the school classroom during class time. It took between 35 and 45 min to complete the scales. All questionnaires were completed individually. If some children could not understood the questions, the researchers read and explained the questions to them. After removing incomplete questionnaires, 401 questionnaires were analyzed.

### Data analysis

To test the hypotheses SPSS version 21 and AMOS version22 were used. Since the factors of the questionnaire were predetermined in the literatures and theoretical model of Ayers and Sandler’s [11], To evaluate the construct validity, confirmatory factor analysis was used. Confirmatory factor analysis aims to measure the fit of the data to a hypothesized determined model [39]. In other words, confirmatory factor analysis seeks to determine whether the number of factors and the loads of variables on these factors correspond to what was expected based on the theoretical model. This type of factor analysis tests the degree of conformity and consistency between the theoretical structure and the experimental structure of the research. Convergent validity was used to assure that the parameters effectively reflect their corresponding factor [40] and the correspondences have to be in a high proportion of variance or among each other [41]. The relationship between subscales was also calculated through the correlations between subscales. The two main methods of internal consistency and item-rest correlations (IRC) were also used to assess the reliability of the questionnaire. Finally, repeated measure ANOVA was also used to assess the status of coping strategies used by the children.

## Results

### Characteristics of participants

The mean age and its deviation for female students who faced earthquake trauma was 11.06 ± 1.28 years. The religion of 52.9% (212 people) was Shiite Islam, 35.4% (142 people) were Sunni Muslims, and 10.5% (42 people) were from Yarsan or Ahl–e-Haq. In terms of education level, 10.5% (42 people) of fathers were illiterate, 42.1% (169 people) had high school and lower than high school degrees, 24.9% (100 individuals) completed high school, and 21.7% (87 people) bachelor’s or a higher degree. In a similar way, 14% (56 people), 47.9% (192 people), 21.2% (85 people), and 16.2% (65 people) of mothers had no literacy, lower high school and lower than high school degrees, completed high school, and bachelor’s or a higher degree respectively. In terms of paternal employment status, 2% (8 people) fathers deceased, 5.5% (22 people) were unemployed, 24.6% (99 people) workers, 20.9% (84 people) clerks, 2.7% (11 people) retired, and 43.3% (174 people) self-employed. Similarly, with maternal 7% (3 people) mothers deceased, 84.8% (340 people) were homemakers, 3.7% (15 people) were workers, 8% (32 people) were clerks, and 2.7% (11people) were self-employed.

### Factor structure

**Fig. 1:**
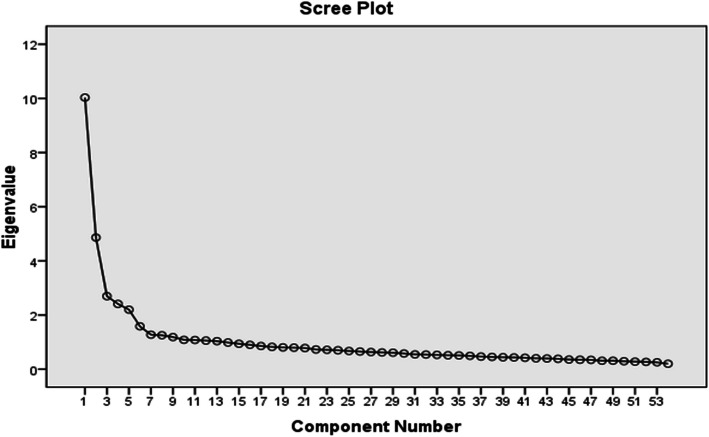
Scree plot of factor loading

Prior to confirmatory factor analysis, Kaiser-Meyer-Olkin (KMO) and Bartlett’s Test of Sphericity were used to determine sampling adequacy and data suitability for the factor analysis. The data analysis indicated sufficient sample size as well as the capability of the variables in factor analysis (KMO = 0.87, x^2^ = 7.72, df = 1431). A scree test was also used to confirm the number of factors in the questionnaire (Fig. 1). According to the scree plot and the eigenvalues above 1, the number of 13 items can be extracted. The total variance explained is 57.78% where the total test variance accounted for every 13 items is between 0.46 and 0.74.

#### Confirmatory factor analysis

The Maximum Likelihood (ML) method was used to estimate the model. In the first stage, the fitting indices of the four-factor and five-factor models were compared and the results are displayed in Table 2; this confirmed that, both the four and the five-factor models are in good agreement with each other. However, following Ayers’ model [42] and the principle of parsimony [43], the four-factor model was preferred and applied in this study.

**Table 2:** Fit indices for each Confirmatory Factor Analysis model tested

Model CCSC-R1	χ2	Df	P	NFI	CFI	SRMR	RMSEA (90% CI)	χ2/df
**Model 1: 4 factor**	103.33	54	0.001	0.96	0.97	0.048	0.04	1.91
**Model 2: 5 factor**	107.8	55	0.001	0.96	0.97	0.048	0.04	1.96

The overall fitness of the model showed that the value of the chi-square to df ratio is higher than the standard value of 3 (χ2/df = 3.08). By applying five modification indices, the fitness index improved to the desired level. The chi-square to df ratio equaled 1.96, indicating an appropriate fit for the model. Other indices of this model, Normed Fit Index or Tucker-Lewis Index (NFI / or TLI = 0.96), Incremental Fit Index (IFI = 0.97), Comparative Fit Index (CFI = 0.97), and Goodness of Fit Index (GFI = 0.94) were higher than the conventional value of 0.9. Also, Root Mean Square Error of Approximation was ideally obtained (RMSEA = 0.04). Regarding these results, the four-factor model demonstrated acceptable results for “absolute fit indices”, “parsimony fit indices”, and “comparative fit indices”. Therefore, the obtained model is adequate for evaluating the target research population, that is, the structural validity is confirmed.

**Fig. 2:**
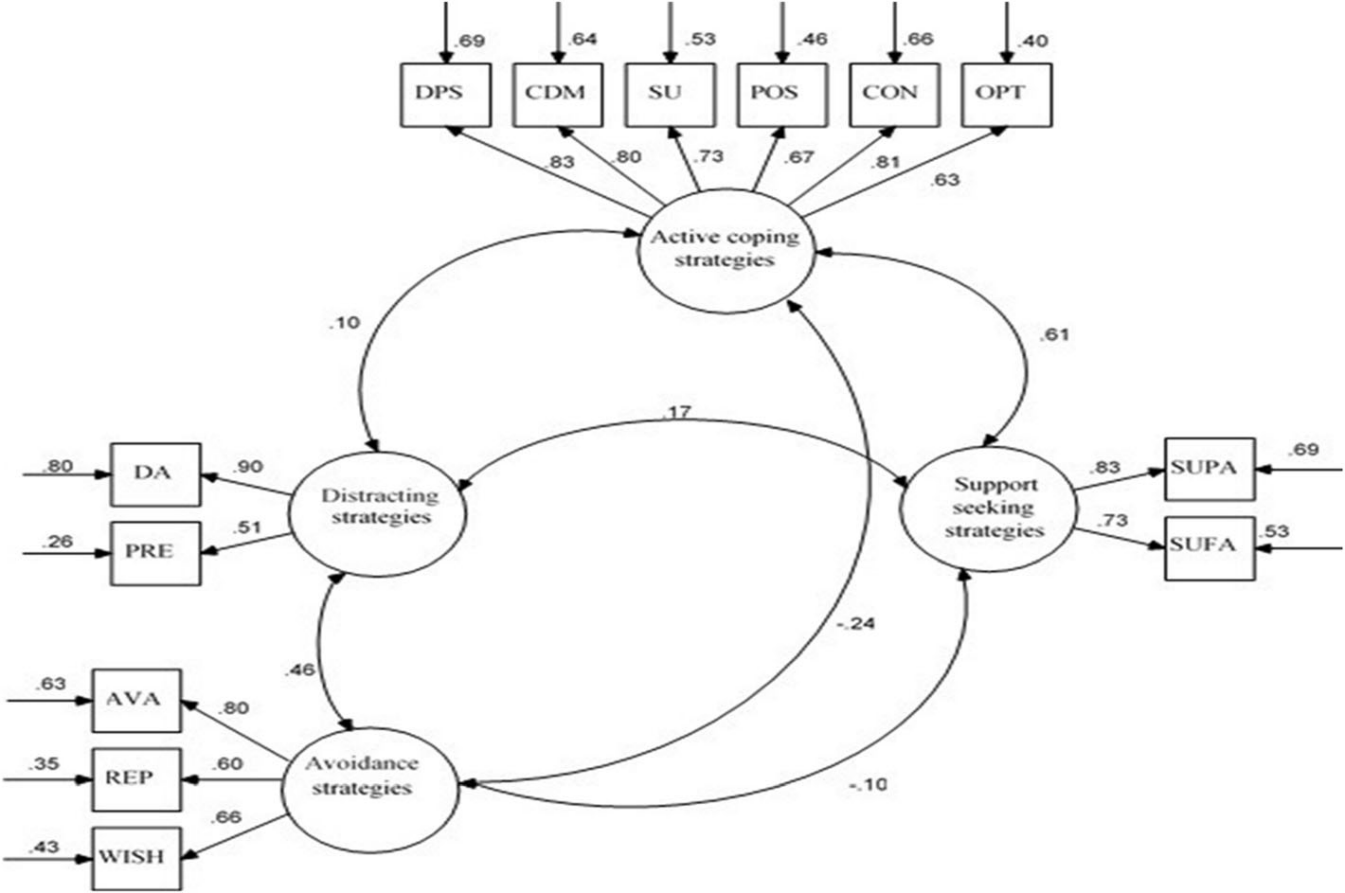
Four-factor structural model equation of the CCSC-R1

Figure 2 shows the structural model equation with the regression coefficients between hidden dimensions and visible indicators model parameters. The range of standard regression coefficient amounts is between 0.51 and 0.90 and the correlation coefficients are between 0.10 and 0.61. The measurement error coefficients are in the range of 0.26 to 0.85. According to this figure, the dimensions have a suitable link with the indicators which confirmed the structural validity. There are two central relationships between dimensions; that is, some of the correlations are strongly significant with a positive direction which indicates correlated dimensions, while, others are both weak and not significant, or possibly have a negative direction, which indicates uncorrelated dimensions.

### Convergent validity

Table 3 demonstrates the validity of CCSC-R1. It confirms that all of the four factors and most subscales have a significant relationship with each other (*p* < 0.01). Also, all of the subscales except Repression (REP) have a strong correlation with CCBQ indicating the convergence validity of the questionnaire.

**Table 3:** Correlation coefficients between CCSC-R1 and CCBQ

***CCSC-R1***	1	2	3	4	5	6	7	8	9	10	11	12	13	14	15	16	17	18	19	20	21
1. active coping	1																				
2.cognitive decision making (CDM)	0.75^**^	1																			
3.direct problem solving (DPS)	0.77^**^	0.68^**^	1																		
4.seeking understanding (SU)	0.65^**^	0.61^**^	0.64^**^	1																	
5.posivity (POS)	0.63^**^	0.37^**^	0.54^**^	0.45^**^	1																
6.control (CON)	0.75^**^	0.56^**^	0.64^**^	0.55^**^	0.58^**^	1															
7.optimism (OPT)	0.62^**^	0.32^**^	0.49^**^	0.38^**^	0.52^**^	0.54^**^	1														
8.distraction strategies	0.31^**^	0.25^**^	0.22^**^	0.13^*^	0.34^**^	0.21^**^	0.17^**^	1													
9.distraction actions (DA)	0.27^**^	0.04	0.05	0.02	0.12^*^	0.09	0.04	0.87^**^	1												
10.physical release of emotions (PRE)	0.26^**^	0.04	0.11^*^	0.04	0.16^**^	0.06	0.03	0.82^**^	0.45^**^	1											
11.avoidance strategies	0.31^**^	0.23^**^	0.23^**^	0.13^*^	0.27^**^	0.23^**^	0.22^**^	0.36^**^	0.33^**^	0.28^**^	1										
12.avoidance action (AVA)	0.28^**^	−0.06	−0.08	−0.074	−0.08	−0.09	−0.04	0.32^**^	0.35^**^	0.19^**^	0.78^**^	1									
13.repression (REP)	0.20^**^	− 0.20^**^	−0.20	−0.34^**^	0.06	0.12^*^	0.02	0.26^**^	0.18^**^	0.14^**^	0.70^**^	0.49^**^	1								
14wishful thinking (WISH)	0.19^**^	−0.21^**^	− 0.17^**^	− 0.15^**^	− 0.19^**^	−0.24^**^	−0.16^**^	0.18^**^	0.27^**^	0.13^**^	0.65^**^	0.52^**^	0.36^**^	1							
15.support seeking strategies	0.47^**^	0.30^**^	0.32^**^	0.30^**^	0.36^**^	0.39^**^	0.29^**^	0.26^**^	0.28^**^	0.16^*^	0.20^**^	0.08	0.19^**^	0.17^**^	1						
16.support for actions (SUPA)	0.45^**^	0.32^**^	0.42^**^	0.39^**^	0.37^**^	0.43^**^	0.34^**^	0.24^**^	0.12^*^	0.02	0.20^**^	−0.08	−0.05	−0.08	0.92^**^	1					
17.support for feelings (SUPF)	0.39^**^	0.30^**^	0.37^**^	0.33^**^	0.31^**^	0.34^**^	0.31^**^	0.23^**^	0.13^**^	0.04	0.17^**^	−0.02	−0.00	0.01	0.87^**^	0.60^**^	1				
***18.Children’s Coping Behavior Questionnaire***	0.55^**^	0.53^**^	0.50^**^	0.48^**^	0.46^**^	0.51^**^	0.47^**^	0.53^**^	0.34^**^	0.30^**^	0.42^**^	0.13^*^	− 0.009	0.05	0.48^**^	0.47^**^77^**^	0.43^**^	1			
19.diversion	0.55^**^	0.54^**^	0.52^**^	0.44^**^	0.49^**^	0.49^**^	0.48^**^	0.53^**^	0.31^**^	0.32^**^	0.40^**^	0.11^*^	−0.01	− 0.03	0.41^**^	0.4^**^	0.38^**^	0.9^**^	1		
20.ameliorative coping	0.52^**^	0.58^**^	0.56^**^	0.57^**^	0.47^**^	0.55^**^	0.43**	0.29**	0.15**	0.12^*^	0.23^**^	−0.06	−0.24^**^	−0.09	0.52^**^	0.51^**^	0.47^**^	0.82^**^	0.66^**^	1	
21.destructive coping	0.15^*^	−0.07	− 0.08	− 0.00	−0.02	0.02	0.04	0.34**	0.30**	0.19^**^	0.33^**^	0.26^**^	0.27^**^	0.34^**^	0.21^**^	0.12^*^	0.077	0.46^**^	0.14^**^	0.17^**^	1

### Reliability

#### Internal consistency

The Cronbach’s Alpha accounts for the four dimensions and subscales in the range of 0.76 to 0.91 and 0.57 to 0.76, respectively (Table 4). Distracting Strategies have a lower alpha (0.76) and Active Coping has the highest one (0.91).

**Table 4:** Internal Consistency of CCSC-R1

	Cronbach’s Alpha	M Inter-item Correlation	Range Corrected Item – Total Correlations
**CCSC-R1**			
**Active coping**	0.91	0.29	0.08–0.52
Cognitive decision making (CDM)	0.71	0.38	0.33–0.45
Distract problem solving (DPS)	0.75	0.43	0.38–0.49
Seeking understanding (SU)	0.72	0.39	0.32–0.44
Posivity (POS)	0.57	0.25	0.17–0.38
Control (CON)	0.76	0.45	0.41–0.50
Optimism (OPT)	0.64	0.32	0.23–0.37
**Distracting strategies**	0. 76	0.29	0.10–0.68
Distraction actions (DA)	0.61	0.26	0.10–0.40
Physical release of emotions (PRE)	0.68	0.35	0.21–0.47
**Avoidance strategies**	0.78	0.24	0.10–0.60
Avoidance action (AVA)	0.58	0.25	0.09–0.41
Repression (REP)	0.68	0.35	0.38–0.53
Wishful thinking (WISH)	0.68	0.35	0.20–0.60
**Support seeking**	0.79	0.32	0.21–0.50
Support for actions (SUPA)	0.73	0.35	0.27–0.42
Support for feelings (SUPF)	0.72	0.39	0.33–0.46

#### Item-rest correlation (IRC)

Using Pearson’s correlation coefficient, allowed us to determine whether the questions were related to the subscales effectively. This coefficient ranged from 0.09 to 0.68 for CCSC-R1 subscales. Almost all of the coefficients obtained from the correlation of the material set were more than 0.3, indicating a moderate to good correlation and a desired internal consistency among subscales (Table 4).

### Status of coping strategies in children

To identify coping strategies used by children, repeated measurement analysis of variance was used. Testing the assumption of normality for the Kolmogorov-Smirnov test showed that the distribution of scores on coping strategies was normal (*p* > 0.05). According to the result of Mauchly’s test (0.31) and given chi-square (459.36) (*P* < 0.001), the assumption of sphericity was not confirmed and Greenhouse-Geisser degrees of freedom were modified to report F-value. Based on the results of Table 5, there is a significant difference between coping strategies used by children (F = 2726.92, p < 0.01, Partialη2 = 0.87).

**Table 5:** Means, standard deviation and results of repeated measurement analysis in children’s coping strategies

Types of coping strategies	Mean	Standard deviation	Source	Sum of Squares	df	Mean square	F	Sig.	Partial Eta (ηp^**2**^)	Observed power
**Active coping**	63.34	13.48	**Greenhouse-Geisser**	546,357.403	1.68	324,009.476	2726.92	0.000	0.87	1
**Distracting strategies**	16.74	5.69								
**Avoidance strategies**	28.67	6.82								
**Support seeking**	20.3	5.8								

**Fig. 3:**
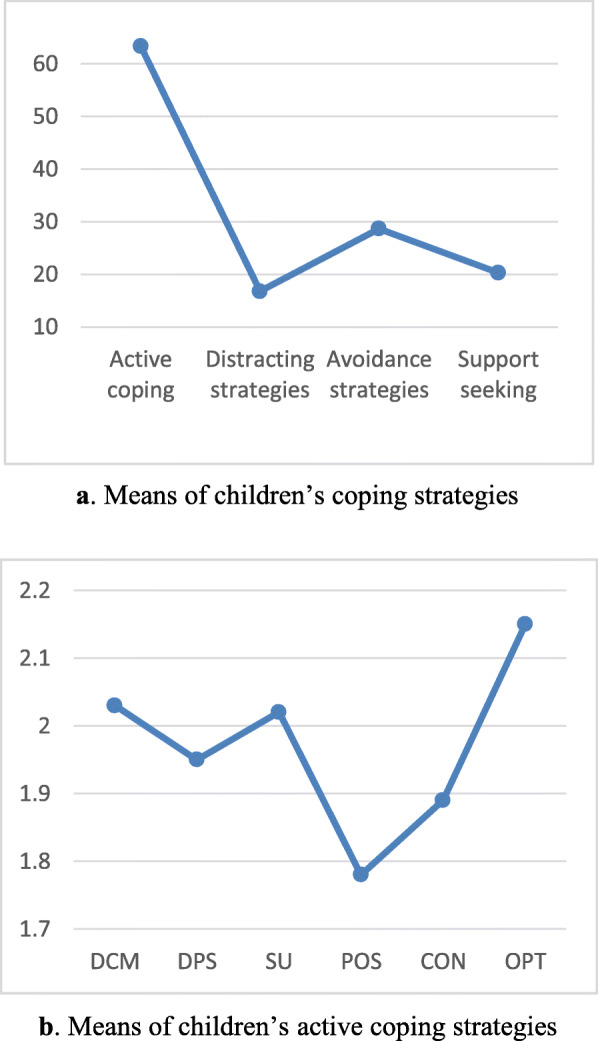
**a***.* Means of children’s coping strategies. **b** Means of children’s active coping strategies

The two-by-two difference between subscales in Table 6 shows that there are significant relations between all four types of strategies. Regarding the mean scores, it can be said that active strategies are the most used when facing earthquake trauma by Iranian adolescent girls. After that, avoidance and support seeking strategies respectively are in the second and third places and distraction strategies are the least used (Fig. 3a).

**Table 6:** Tukey’s multiple comparison test of children’s coping strategies

	Mean difference (I-J)	Standard error	Sig.
**Active coping- Support seeking**	43.03	0.58	0.0001
**Active coping- Distracting strategies**	46.59	0.7	0.0001
**Active coping- Avoidance strategies**	34.66	0.61	0.0001
**Support seeking- Distracting strategies**	3.56	0.38	0.0001
**Avoidance strategies- Support seeking**	8.37	0.46	0.0001
**Avoidance strategies-Distracting strategies**	11.93	0.36	0.0001

Repeated measurement analysis of variance was also used to investigate which active coping strategies were most commonly used in children. Due to the unconfirmed assumption of sphericity and significance of Mauchly’s test (0.92) and given chi-square (32.76) (P < 0.01), Greenhouse-Geisser were modified to report F-value. According to Table 7, there is a significant difference between the six active coping strategies used by children (F = 2726.92, *p* < 0.01, Partialη2 = 0.87).

**Table 7:** Means, standard deviation and results of repeated measurement analysis in children’s active coping strategies

Types of active coping strategies	Mean	Standard deviation	Source	Sum of Squares	df	Mean square	F	Sig.	Partial Eta (ηp^**2**^)	Observed power
Cognitive decision making (CDM)	2.03	0.03	**Greenhouse-Geisser**	32.577	4.841	6.729	32.475	0.000	0.075	1
Distract problem solving (DPS)	1.95	0.02								
Seeking understanding (SU)	2.02	0.03								
Posivity (POS)	1.78	0.02								
Control (CON)	1.89	0.03								
Optimism (OPT)	2.15	0.03								

All of the active coping strategies subscales (except cognitive decision making and seeking understanding) significantly different from each other. Mean scores show that optimism is the most active coping strategy that children use when faced with problems (Table 8). Then, cognitive decision making, seeking understanding, Distract problem solving, Control and Posivity were used respectfully by Iranian children (Fig. 3b).

**Table 8:** Tukey’s multiple comparison test of children’s active coping strategies

	Mean difference (I-J)	Standard error	Sig.
DCM-DPS	0.075	0.02	0.009
DCM-SU	0.013	0.03	0.6
DCM-POS	0.248	0.03	0.000
DCM-CON	0.138	0.03	0.000
DCM-OPT	−0.125	0.03	0.000
DPS-SU	−0.062	0.02	0.03
DPS-POS	0.173	0.03	0.000
DPS-CON	0.062	0.03	0.03
DPS-OPT	−0.200	0.03	0.000
SU-POS	0.235	0.03	0.000
SU-CON	0.125	0.03	0.000
SU-OPT	−0.138	0.03	0.000
POS-CON	− 0.110	0.03	0.001
POS-OPT	−0.373	0.03	0.000
CON-OPT	−0.263	0.03	0.000

## Discussion

The purpose of this study was to investigate the psychometric properties of the CCSC-R1, as a proposed tool to provide an applied inventory for measuring the coping strategies of Iranian 9–13-year-old children. The data were collected from 401 children in Sarpol-e-Zohab region (Iran) who experienced an earthquake.

The results of the confirmatory factor analysis on experimental data showed the fitness of the four-factor conceptual model in the population of Iranian children. These results coincide with the theoretical models of Ayers et al. [12], which proposed a four-factor model that can be useful for both the situation-specific and dispositional coping. The Cronbach’s alpha, in the range of 0.76 to 0.91 indicates an appropriate internal consistency and reliability of CCSC-R1. Convergence validity was also confirmed by the optimal correlation between CCSC-R and CCBQ tests. Comparing the range of correlation coefficients obtained for the scores on 13 components of CCSC-R1 and the scores on four factors indicates that four factors of CCSC-R1 can be conceptualized and distinguished from each other.

The findings of this study are consistent with all previous studies confirming the validity and reliability of CCSC-R1 including 13 subscales [11, 17–23, 44]. The research findings on the classification of subscales into four factors are similar to those of Ayers & Sandler [11], Scott [17] and Thorne et al. [20] who classified 13 subscales of CCSC-R1 questionnaire into four categories including active, avoidance, distraction, and support seeking coping strategies. On the other hand, these findings are inconsistent with the results of Camisasca et al. [22] and de Boo & Wicherts [21]. These studies were respectively conducted in Italy and the Netherlands and the items included in subscales were assigned into five factors: problem-focused, positive cognitive restructuring, avoidance, distraction, and social support seeking coping strategies. Given the similarity of subscales including 13 items in all studies, the difference in the number of associated factors can be attributed to the implementation method, sample size, or the degree of homogeneity of participants.

The above results prove that the typology based on Ayers’ theoretical model on children’s coping strategies can be generalized to Iranian children’s culture and norms with caution. In other words, coping strategies in Iranian children are conditioned by more or less four factors.

Also, in line with Samaraweera [45] who stated that people use different coping ways when dealing with natural disasters, the study of the status of coping strategies used by adolescents showed that in the face of earthquake trauma, all active coping, distraction, avoidance, and support-seeking strategies are used by Iranian adolescents. Active-behavioral coping defines as behavioral attempts to deal with a problem directly [46] (e.g., cognitive decision making, distract problem solving, seeking understanding, posivity, control, and optimism). Distraction defines as behaviors or thoughts (e.g., listening to music, watching TV, riding bicycle, and walking) that take the person’s mind off the problem and reduce the impact of negative mood on information processing and memory [47]. Avoidance coping strategies are the activities and/or cognitive strategies (e.g., wishful thinking, repression, tried to stay away from the problem, and feel upset) used in an intentional attempt to disengage from stressful situations [48]. These strategies tend to reduce distress and anxiety in the short run, soon after the stressful situation occurs (within a week). However, they are less adaptive to be psychological well-being for the long-term [49]. Support seeking coping includes strategies such as talking about the feelings to someone who really understand the situation, let other people know about your feelings, telling people what they should do for you, talking to someone who can help you to solve the problem. Generally, support seeking coping has two kinds: Action and feeling support seeking. Duhachak [50] defined Emotional/or feeling support-seeking coping behaviors as “attempts to marshal social resources to improve one’s emotional and/or mental state.” Consumers coping in this manner “seek out others for comfort.” (p. 44) and instrumental/or action support seeking is defined as “attempts to marshal social resources to take action towards ameliorating a stressor,” (p. 46) coping which includes co-opting the assistance of social resources with the intent of the stress situation moderation directly. The difference between this coping strategy and emotional support is its focus on bringing objective change. The individual attempts to get advice about his/her situation from someone else to find what to do [50].

However, contrary to the assumption that problem-solving, self-distancing from the problem [27], distraction, and avoidance strategies are expected from people of collectivist cultures to control and suppress their emotions and behaviors [51], Iranian children rather used active strategies to cope with the trauma. Among active coping strategies, optimism is the widely used method by Iranian children when they face a problem. This means that children tend to consider the most hopeful perspective when they face their problems and cope with it. They usually expect positive outcomes, which are considered to be constant, general, and internal factors [52]. Considering these consequences, they get to the point at an emotional and cognitive premise which good things are more important than bad things [53].

Explaining this finding, it can be said that Iranian education has been teaching life skills and the ability to select and make decisions, think, explore, judge, and evaluate in primary schools for several years. Learning these skills will help children strive for solutions. They will learn to use active strategies, gather information about the stressful event, think about it with an optimistic view, and plan to use their available resources [54].

Moreover, it seems that Iranian adolescent girls behave in a similar way to people in societies with individualistic cultures in the face of trauma. People of individualistic cultures are more outspoken and honest in conversations or conflicts and would rather use active strategies [55]. It was shown that Iranian adolescents like Europeans (Croatia, the Czech Republic, Germany, Italy, Norway, Portugal, and Switzerland) used active coping strategies to address future-related problems [56] and post-earthquake problems. Although scant research has been done on coping strategies of children and adolescents in the Middle East, in accordance with the findings of this study, supported by the reports of Braun-Lewensohn et al. [33] and Shirazi et al. [34], it can be said that Middle Eastern adolescents act rather similar to people in Western and American countries with an individualistic culture compared to Asian countries with a collectivist culture when coping with traumatic events. Further cultural studies are required to confirm this claim.

## Recommendations for future research

In order to further examine the psychometric characteristics, other tests of validity and reliability such as content validity, construct divergent validity, test-retest reliability can be used. Future studies on larger groups in different cities of Iran will also help to develop the questionnaire. Since only Iranian adolescent girls were included in the present study, adolescent boys and gender differences should be considered in future research. Indicating common cultural factors, the similarity between Iranian, Western, and American adolescents in the use of active strategies provides the ground for extensive cross-cultural studies.

### Limitations of the study

Since gender is an important factor in the use of coping strategies, the generalization of results to the community of Iranian girls should be applied with caution. When citing the results from our research, it should be noted that the use of self-reporting instruments will not provide accurate information. To evaluate construct validity, only one questionnaire was used. This is mainly because our participants were young and might become tired of answering many questionnaires.

## Conclusions

While culture plays an undeniable role in the coping strategies and reactions of individuals to stressful situations [57] findings of this study are consistent with previous results of investigations on American, African-American, European-American, Latino, Biracial, Asian-American, Canadian, Dutch, and Italian children. This result shows that the proposed coping strategies by Ayers [42] are independent of culture and can be considered for understanding the coping strategies of children, regardless of their culture and race Although the number of subscales (13 items) were constant, the main reasons for difference in the number of factors were the method of implementation, the number of participants, and the homogeneity of the sample which may need replication.

The results of the present study provide preliminary empirical support for measuring coping strategies in Iranian children.. Our study results also provides a platform for further steps to be performed in this area and further use of CCSC-R1 coping strategies in basic and applied research, including therapeutic studies. Given that Iran is among one of the most earthquake-prone countries, knowing that active coping, especially optimism, is the most frequently used strategy by Iranian adolescent girls in the face of earthquake trauma, may provide grounds for psychological support mechanisms to support them in coping with natural disasters..

## Data Availability

The datasets generated and/or analyzed during the current study will be available from the corresponding author upon reasonable request.

## Abbreviations

NA: Not Available
KMO: Kaiser-Meyer-Olkin
NFI: Normed Fit Index
TLI: Tucker-Lewis Index
IFI: Incremental Fit Index
CFI: Comparative Fit Index
GFI: Goodness of Fit Index
RMSEA: Root Mean Square Error of Approximation
